# Expression of deleted, atoxic atypical recombinant beta2 toxin in a baculovirus system and production of polyclonal and monoclonal antibodies

**DOI:** 10.1186/s12934-017-0707-8

**Published:** 2017-05-25

**Authors:** Anna Serroni, Chiara Francesca Magistrali, Giovanni Pezzotti, Luca Bano, Martina Pellegrini, Giulio Severi, Chiara Di Pancrazio, Mirella Luciani, Manuela Tittarelli, Silvia Tofani, Antonio De Giuseppe

**Affiliations:** 1 Istituto Zooprofilattico Sperimentale dell’Umbria e delle Marche, Via G. Salvemini 1, 06126 Perugia, Italy; 2 Istituto Zooprofilattico Sperimentale delle Venezie, Vicolo Mazzini 4, Villorba Di Treviso, Italy; 3 Istituto Zooprofilattico Sperimentale dell’Abruzzo e del Molise “G.Caporale”, Teramo, Italy; 4 Scuola di Specializzazione “Biochimica Clinica” G. d’Annunzio, University Chieti-Pescara, Chieti, Italy

**Keywords:** Atoxic rCPB2_Δ1–25_-His_6_, Ni^2+^ affinity chromatography, Monoclonal antibody

## Abstract

**Background:**

*Clostridium perfringens* is an important animal and human pathogen that can produce more than 16 different major and minor toxins. The beta-2 minor toxin (CPB2), comprising atypical and consensus variants, appears to be involved in both human and animal enterotoxaemia syndrome. The exact role of CPB2 in pathogenesis is poorly investigated, and its mechanism of action at the molecular level is still unknown because of the lack of specific reagents such as monoclonal antibodies against the CPB2 protein and/or the availability of a highly purified antigen. Previous studies have reported that purified wild-type or recombinant CPB2 toxin, expressed in a heterologous system, presented cytotoxic effects on human intestinal cell lines. Undoubtedly, for this reason, to date, these purified proteins have not yet been used for the production of monoclonal antibodies (MAbs). Recently, monoclonal antibodies against CPB2 were generated using peptides designed on predicted antigenic epitopes of this toxin.

**Results:**

In this paper we report, for the first time, the expression in a baculovirus system of a deleted recombinant C-terminal 6xHis-tagged atypical CPB2 toxin (rCPB2_Δ1–25_-His_6_) lacking the 25 amino acids (aa) of the N-terminal putative *signal sequence*. A high level of purified recombinant rCPB2_Δ1–25_-His_6_ was obtained after purification by Ni^2+^ affinity chromatography. The purified product showed no in vitro and in vivo toxicity. Polyclonal antibodies and twenty hybridoma-secreting Mabs were generated using purified rCPB2_Δ1–25_-His_6_. Finally, the reactivity and specificity of the new antibodies were tested against both recombinant and wild-type CPB2 toxins.

**Conclusions:**

The high-throughput of purified atoxic recombinant CPB2 produced in insect cells, allowed to obtain monoclonal and polyclonal antibodies. The availability of these molecules could contribute to develop immunoenzymatic methods and/or to perform studies about the biological activity of CPB2 toxin.

## Background

*Clostridium perfringens* is an anaerobic, gram-positive, spore-forming bacterium distributed ubiquitously in the environment and the gut of many healthy humans and animals. This bacterium can cause several diseases due to the various toxins that it produces [1]. *Clostridium perfringens* strains are classified into five different types (A-E) based on the expression of four major toxins—alpha, beta, epsilon and iota toxins—each of which determines a specific pathogenicity [2–5]. In addition to the four major toxins, some *C. perfringens* strains can express other different toxins, defined as minor toxins, such as β2-toxin (CPB2), which was first identified in a *C. perfringens* strain isolated from a piglet. The designation β2-toxin is motivated by the pathogenic activities, similar to β-toxin, despite the poor genetic homology between the two toxins [4–6].

The *cpb2* gene is located on a large plasmid of *C. perfringens* encoding a mature 28-kDa-molecular-weight toxin, which is secreted in the cellular culture supernatant essentially in the sporulation phase [6]. The CPB2 protein is encoded by two different alleles—the consensus gene allele and atypical gene allele—which encode a less toxic CPB2 variant [7–9]. However, the presence of the *cpb2* gene is not necessarily associated with the expression of the protein because in some *cpb2*+ *C. perfringens* strains, no presence of the toxin was observed. It has been reported that the loss of atypical CPB2 synthesis was due to a frame-shift mutation at position 178 in the *cpb2* gene [7]. As observed in the expression of alpha, kappa and theta toxins in C*. perfringens*, the transcription of the *cpb2* gene is regulated by the two-component VirR and VirS system and its secondary RNA regulator VR-RNA [5, 10, 11].

Since the CPB2 toxin has been identified, many studies have reported its presence in *C. perfringens* strains isolated in enteritis and enterotoxaemia of many animal species, including humans [8, 12–16]. The atypical *cpb2* gene is more frequently found in non-porcine isolates of *C. perfringens* [7]. Consensus and atypical CPB2 proteins show a 62% amino acid identity and an 80% similarity, whereas atypical CPB2 toxins were from 96.2 to 98.9% identical and 97 to 99.2% similar to each other [6, 7].

The pathogenic role of CPB2 toxin is still debated. Indeed, in some species such as piglets, there seems to be a clear correlation between the lesions and presence of CBP2; however, in other species, including chickens and humans, this relationship has not been confirmed. Further studies demonstrated the in vitro cytotoxicity of the native CPB2 toxin in different intestinal cell lines; however, currently, its exact role in pathogenesis remains unclear [13, 15–20]. In the last decades, many studies have been focused on the use of purified recombinant consensus CPB2 expressed in prokaryotic systems for immunological and crystallographic studies [21, 22]. Moreover, recombinant consensus CPB2 protein was employed as an antigen to produce antibodies and was subsequently used for the development of an Enzyme-linked Immunosorbent Assay (ELISA) for the detection and quantification of the toxin in the piglet’s intestinal material [6, 7, 9, 13, 23–25].

However, the lack of purified CPB2 and specific MAbs has limited the possibility to investigate the pathogenic mechanisms, biological function of the CPB2 toxin and development of an efficient immunoenzymatic assay. Recently, Zeng et al. showed that purified recombinant His-tagged CPB2 toxin, produced in *E. coli,* could induce apoptosis in NCM460 cells. Notably, in their study peptides designed on predicted antigenic epitopes were employed to generate MAbs against CPB2. The functionality of the three MAbs obtained was evaluated in their capacity to neutralize the cytotoxicity of recombinant CPB2 and in immunoreactions such as immunoblots, immunofluorescence and ELISA [26].

In this work, we report for the first time, the expression of non-toxic atypical CPB2 toxins, lacking the putative *signal sequence*, in a baculovirus system and its use as an antigen for the production of polyclonal and monoclonal antibodies. Immunoenzymatic tests were used to evaluate the reactivity and specificity of the new MAbs against atypical and consensus proteins produced by field strains of *C. perfringens*.

## Results

### Expression and analysis of recombinant β2 toxins

**Fig. 1:**
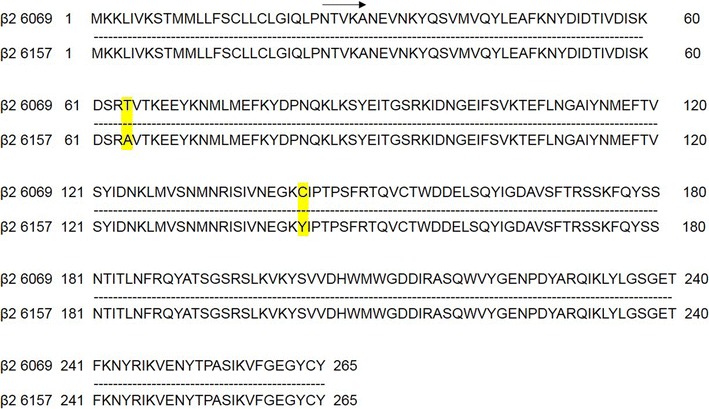
Comparison of the amino acid sequence of the protein β2 between the atypical isoforms 6069 and 6157. The mutation changes in the amino acid sequence between the two proteins are highlighted in *yellow*

The nucleotide sequence corresponding to the putative *signal sequence* encoding the first 25 amino acids of the amino terminus of the atypical *cpb2* gene [6, 7] was removed by PCR. The full-length and 5′ deleted *cpb*2 genes encoding atypical enterotoxins clones 6069 and 6157 were inserted *in frame* with a 6xHis-tag into baculovirus expression vectors pOET2_C-6xHis (Oxford Technologies Expression) to generate pOET-β2His/6069, pOET-β2His/6069Δ_1–25_, pOET-β2His/6157 and pOET-β2His/6157Δ_1–25_ constructs. The amino acid sequence derived from the amplified nucleotide sequences showed a 99% homology between the two atypical forms. Nevertheless, the two strains were isolated from the same region, and a change of two amino acids (aa positions 64 and 143) was observed in the amino acid sequence (Fig. 1).

**Fig. 2:**
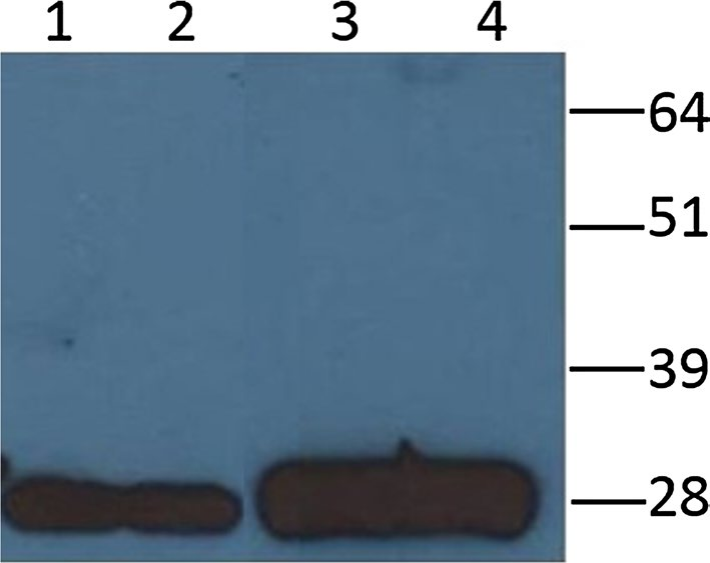
Recombinant atypical beta2 toxin characterization by Western blotting using anti-His C-Term MAb. The supernatant of recombinant atypical β2 toxins 6157 and 6069, full-length forms (*lanes 1* and *2*), the cellular lysate of recombinant atypical β2 toxins 6157 and 6069, deleted forms (*lanes 3* and *4*). The molecular weight standard is on the *right*

The proteins were expressed by the infection of Sf21 insect cells with the recombinant baculoviruses and were analysed by Western blotting using the anti-His C-Term HRP monoclonal antibody. The results showed the presence of a specific immune-reactive band with the expected molecular weight of approximately 27–28 kDa for each clone (Fig. 2). Notably, a high level of expression of the antigens was observed for both the intracellular and secreted forms.

The identity of recombinant proteins was further verified using polyclonal rabbit serum (data not shown).

### Purification of recombinant enterotoxins

Recombinant baculovirus bearing atypical 6069 and 6157 deleted genes were compared for their capacity to produce the respective recombinant proteins. As shown in Fig. 2, under the same experimental conditions, the two recombinant baculoviruses produced approximately the same amount of proteins. These data were confirmed after purification by Ni^2+^ affinity chromatography and quantification of both 6069 and 6157 atypical CPB2 (data not shown). Therefore, based on these results, we decided to perform subsequent studies using the deleted recombinant baculovirus expressing clone 6157 (rCPB2Δ_1–25_-His_6_).

**Fig. 3:**
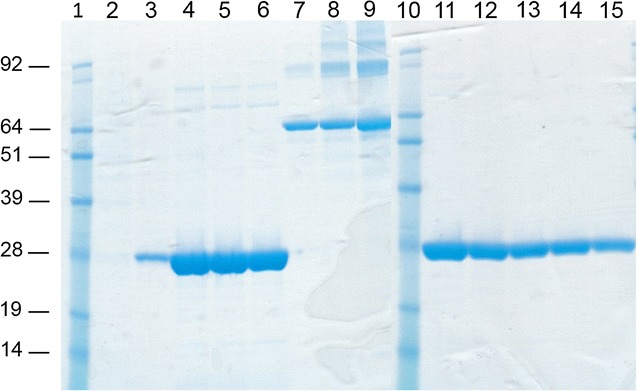
SDS-PAGE and Coomassie gel staining of purified recombinant rCPB2_Δ1–25_-His_6_. Purified recombinant rCPB2_Δ1–25_-His_6_ elution fractions (*lanes 2*–*6* and *11*–*15*); BSA standards, 1 μg (*lane 7*), 2 μg (*lane 8*) and 5 µg (*lane 9*); molecular weight standard (*lanes 1* and *10*)

In the Ni^2+^ affinity purification, different experimental conditions were tested. In addition to the native condition, the protein was also purified under denaturing and reducing conditions in the presence of 8 M urea and 5 mM DTT. The highest yield and best degree of purity were obtained using the procedure performed under denaturing and reducing conditions. A very high recovery with a high degree of purity of protein was obtained using an elution buffer at pH 4.5. Indeed, the quantification of purified product by the Bradford method showed a final yield of approximately 8 mg for 10^9^ insect cells with a degree of purity ≥98% CPB2, as evidenced by staining of the gel with Coomassie (Fig. 3).

### Evaluation of in vitro and in vivo toxicity tests

Prior to the inoculation of the animals, to produce polyclonal and monoclonal antibodies, the purified intracellular atypical rCPB2_Δ1–25_-His_6_ produced in the baculovirus system was tested for its potential in vitro cytotoxicity. In the first approach, the cytotoxic effect of rCPB2_Δ1–25_-His_6_ was evaluated in terms of morphological changes in treated and untreated CaCo-2 cells. No morphological changes were observed after 12 h of incubation of the cells with the rCPB2_Δ1–25_-His_6_ protein even at a concentration of 50 µg/ml (data not shown). The toxicity of this antigen was confirmed by in vivo inoculation. No clinical manifestation was observed after 1 week of inoculation and, in addition, the animals continued to look healthy even after 4 weeks of observation.

### Polyclonal and monoclonal antibodies

To obtain monoclonal and polyclonal antibodies, Balb/c mice and rabbits were immunized with the purified recombinant deleted rCPB2_Δ1–25_-His_6_. The high immunogenicity of recombinant atypical CPB2 in rabbits was evidenced by seroconversion using the iELISA test. Indeed, high OD_450_ values, already after the second inocula, were observed (data not shown).

**Fig. 4:**
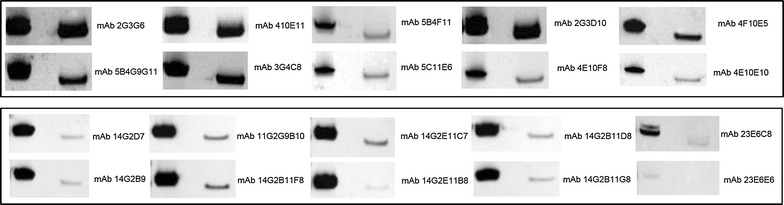
Characterization of monoclonal antibodies. Each MAb was tested against rCPB2_Δ1–25_-His_6_ (*first lanes*) and wild-type CPB2 protein (*second lanes*) produced by *Clostridium perfringens* using the immunoblotting assay

Twenty hybridoma cell lines secreting MAbs versus recombinant atypical CPB2 protein were obtained; to evaluate their reactivity, Western blotting and ELISA were performed. In Western blotting, each MAb recognizes native and recombinant CPB2 protein with an expected MW. Notably, the immunoreactive band of the wild-type CPB2 runs faster than the corresponding recombinant protein. In addition, there was a reactivity difference between the 20 clones analysed. MAbs 2G3G6, 4E10E11, 2G3D10, 5B4G9G11, and 3G4C8 showed strong reactivity with both proteins, whereas MAb 4F10E5 evidenced moderate reactivity. The reactivity of MAbs 14G2D7, 14G2B9, 11G2G9B10, 14G2B11F8, 14G2E11C7, 14G2E11B8, 14G2B11G8 and 14G2B11D8 showed strong reactivity with the recombinant CPB2 and a discrete or faint reactivity with the wild-type protein. The clones 5C11E6, 5B4F11, 4E10F8, 4E10E10 and 23E6C8 showed a lower reactivity with a certain degree of variable between the two proteins; only the hybridoma clone 23E6E6 produced a MAb apparently unreactive with the proteins under denaturing and reducing conditions (Fig. 4).

The results obtained in ELISA apparently showed reactivity against recombinant and wild-type CPB2 of the MAbs 2G3G6, 4E10E11 2G3D10, 5B4G9G11, 3G4C8, 4F10E5, 14G2D7, 14G2B9, 11G2G9B10, 14G2B11F8, 14G2E11C7, 14G2E11B8, 14G2B11G8 and 14G2B11D8, similar to those observed in Western blotting (data not shown).

**Fig. 5:**
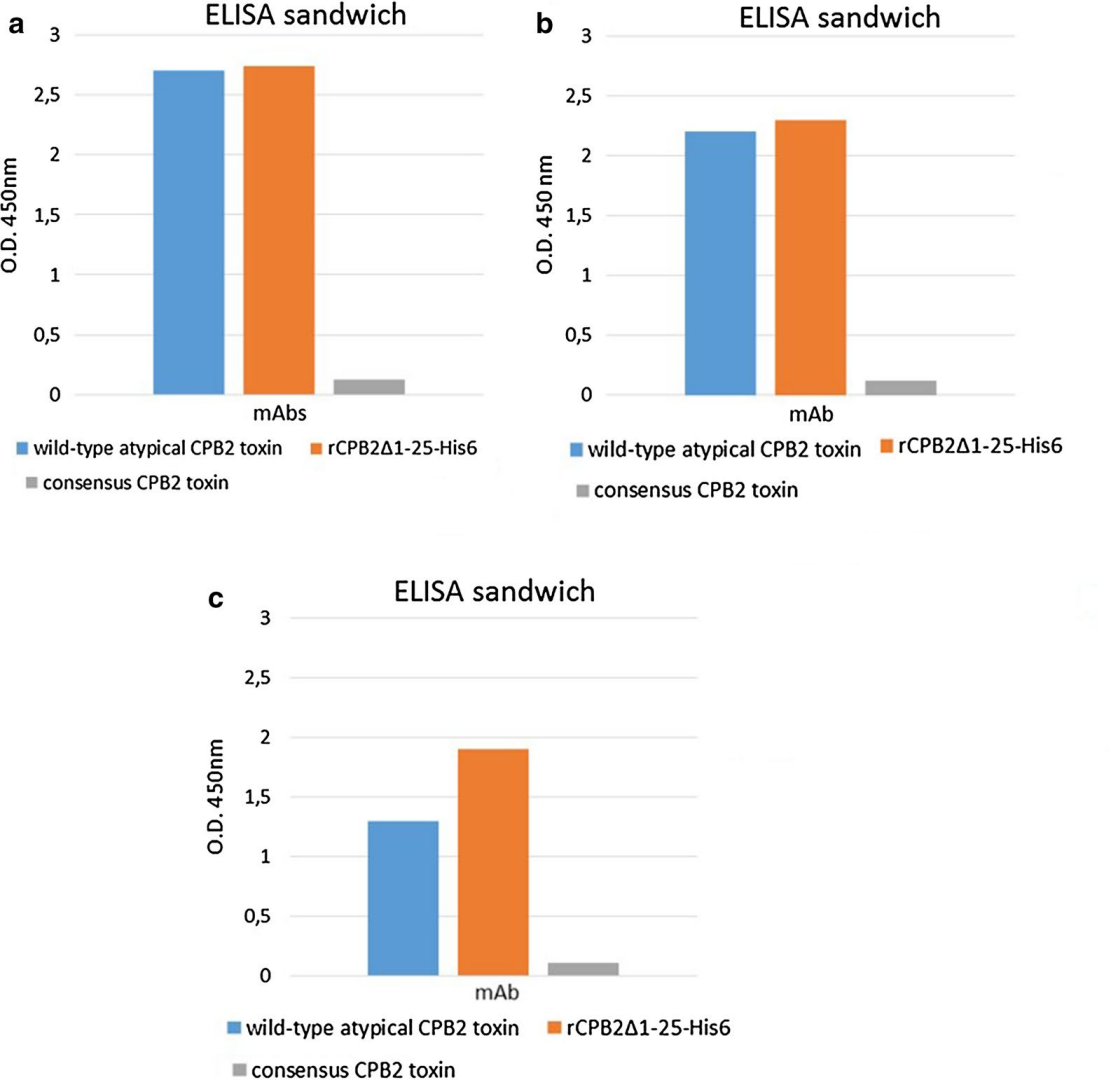
MAbs characterization by ELISA. MAbs 5B4F11, 4E10E10, 5C11E6, 4E10F8 (**a**), 23E6C8 (**b**) and 23E6E6 (**c**) were analysed using wild-type atypical toxin rCPB2_Δ1–25_-His_6_ and consensus CPB2 proteins

Four of the six remaining hybridomas, 5B4F11, 4E10E10, 5C11E6 and 4E10F8, presented higher reactivity in ELISA than in Western blotting (Fig. 5a). In addition, the MAb clone 23E6C8 in Western blotting showed a discrete interaction with the recombinant CPB2 toxin and very faint reactivity with the wild-type protein, but ELISA showed discrete reactivity against recombinant and wild-type beta2 toxin (Fig. 5b). The clone 23E6E6 (which, in Western blotting, presents very low reactivity against recombinant CPB2 and no reactivity with the wild-type toxin) demonstrated good reactivity as indicated by the values of OD_450_ reported in Fig. 5c.

The specificity of the MAbs obtained was verified using consensus CPB2 toxin obtained from genetically consensus *cpb2*+ *C. perfringens* culture as shown in Fig. 5a–c.

## Discussion

Necrotic enteritis induced by *C. perfringens* in humans and animals has spread worldwide. This disease is attributed to the action of major and minor toxins, such as CPB2 toxins [2, 4, 6], produced by this anaerobic bacterium. The information concerning the in vitro cytotoxicity of CPB2 and its involvement in the development of diarrhoea is quite controversial. Previous studies have reported that both consensus and atypical CPB2 enterotoxin are not involved in in vitro cytotoxicity [31]. On the other hand, other studies have indicated that the CPB2 derived from porcine and human *C. perfringens* strains showed dose-dependent cytotoxic activity in human Caco-2 and I407 intestinal cells [5, 6, 18]. Other authors have reported that the minor CPE and CPB2 toxins may act in synergy with the major alpha enterotoxin [6, 24]. In recent reports, Zeng et al. demonstrated that *E. coli*-expressing His-tagged recombinant atypical CPB2 binds the cell membrane and exhibits moderate cytotoxic activity in NCM460 human intestinal epithelial cells [26]. To date, the exact role of CPB2 toxin is unclear, particularly the role of the atypical forms, in both humans and animals. The study of the hypothetical role and biological activity of CPB2 toxin in *C. perfringens* pathogenesis is hampered by the lack of specific reagents such as monoclonal antibodies. For example, the availability of rapid and efficient immunoassays such as ELISA, based on the use of these antibodies, could ensure the identification of the toxin. Furthermore, it could lay the groundwork for investigations/studies of protein biological activity in the intestinal tract and its implication in *C. perfringens*-associated enterotoxaemia and/or whether it represents a simple virulence factor.

In this study, we expressed, for the first time, in a baculovirus system, a high level of non-toxic full-length and a 25-aa-deleted amino-terminal CPB2 atypical enterotoxin of *C. perfringens.* Nucleotide sequence analysis and comparison between the aa sequences of clones 6157 and 6069 revealed, according to other authors [7, 8], an identity of 99%. Figure 1 shows that the variation was present in two amino acids at position 64 (threonine in CPB2-6069 and alanine in CPB2-6157) and 143 (cysteine in CPB2-6069 and tyrosine in CPB2-6157). Considering that both strains were isolated from the same geographical area, in the north of Italy, these data confirmed the high polymorphism characteristic of the atypical CPB2 toxin [7].

Expression studies performed by Western blot analysis showed immune-reactive bands with the expected molecular weight of 28 kDa. Previously, it was reported that the first 30 N-terminal aa of CPB2 enterotoxin represent the putative signal sequence [6, 7, 24, 32]. The presence of the 28 kDa immune reactive band in the culture supernatant but not in the cellular extract of *C. perfringens* strains isolated from pigs with signs of gastrointestinal disease demonstrated that CPB2 was a secreted protein [6, 7, 13]. In our experiments, an immunoreactive 28 kDa band was observed in the Sf21 cell culture supernatant infected with recombinant baculovirus expressing full-length CPB2. By contrast, in the Sf21 cell medium infected with recombinant baculovirus expressing rCPB2_Δ1–25_-His_6_, the corresponding 28 KDa band was absent (data not shown), confirming the role of the *signal sequence* represented by the first 30 aa of the N-terminal region of the toxin. Notably, both extracellular and intracellular recombinant CPB2 proteins showed the same MW, indicating that the signal peptide was removed in the secreted form. The MW of recombinant proteins appeared to be higher than that of wild-type CPB2, indicating that the CPB2 expressed by insect cells could be modified by post-translation events. However, the level of expression of deleted intracellular recombinant CPB2 was higher than that of the secreted form (data not shown). In addition, the best results in terms of the production and quality of purified product were obtained with the atypical clone 6157 when Ni^2+^ affinity chromatography was carried out under denaturing and reducing conditions of the cellular lysate. This feature could be explained by major exposure of the C-terminal 6xHis tag of the denatured antigen with respect to the folded protein. Under these conditions, it was possible to obtain 8 mg of purified recombinant protein per litre of infected Sf21 insect cells with a purity equal to or higher than 98%.

Previous studies have shown that purified recombinant atypical CPB2 was partially toxic to NCM460 cells [26]. Our studies, instead, demonstrated that the full-length and rCPB2_Δ1–25_-His_6_ antigens produced in the baculovirus system were not toxic. Indeed, no changes in the morphological aspects of the CaCo-2 cells were observed in the presence of high concentration of both proteins. In addition, the cells continued to grow, maintaining their classical morphology for a long period of incubation in the presence of the purified recombinant CPB2 proteins. The intravenous minimal lethal dose of the porcine beta2 native toxin for mice was 3 µg [6]; however, in our experiments, no clinical signs were observed in the Balb/C mice and rabbits intraperitoneally inoculated with 100 and 500 µg, respectively, of purified rCPB2_Δ1–25_-His_6_, confirming the harmlessness of the antigen. Another advantage of the baculovirus system to express recombinant atypical CPB2 toxin is that this method ensures a biosafety level higher than that of prokaryotic expression, especially when associated with the easy-to-use and rapid Ni^2+^ affinity chromatography purification method under denaturing and reducing conditions.

Although CPB2 atypical toxin is considered less toxic than the consensus variant [7, 8, 16, 33], the loss of in vitro and in vivo toxicity of our antigens, without any change of antigenic characteristics, could depend on different aspects: (i) the rCPB2_Δ1–25_-His_6_ could be non-toxic when inoculated intraperitoneally, in line with that previously observed in in vitro experiments [31]; (ii) the polymorphism of CPB2 could lead to variable toxicity, if not to a non-toxic protein variant/s; (iii) the CPB2 forms expressed in the baculovirus system, as reported above, could present little post-translation modifications with respect to the wild-type or recombinant CPB2 produced by *E. coli,* which could alter the biological function of these molecules without changes in the antigenic characteristics. This last hypothesis can be supported by the results reported in Fig. 4, where the MW of the rCPB2_Δ1–25_-His_6_ protein is slightly higher than that of the wild-type protein. Further studies are needed to establish this hypothesis, especially for hypothetical future use of a non-toxic antigen as a vaccine.

However, the main advantage of the use of non-toxic recombinant CPB2, produced in insect cells, was to generate polyclonal and monoclonal antibodies by intraperitoneal injection of the purified product. Twenty hybridoma clones producing mAbs were generated, and they could cross-react, except for MAb 23E6E6, with recombinant and native CPB2 using both ELISA and Western blotting. All of the MAbs showed a high specificity because they were unreactive against recombinant and wild-type consensus CPB2 toxin (Fig. 5). The good reactivity of clone 23E6E6 in ELISA and the absence of a clear immunoreactive band in immunoblotting provide evidence that it is a conformational type antibody. The same reactivity of the MAbs 2G3G6, 4E10E11, 2G3D10, 5B4G9G11, 3G4C8 and 4F10E5 versus both the native and recombinant atypical CPB2 observed using Western Blotting and ELISA, demonstrated that the MAbs are direct against a conserved linear epitope in the CPB2 protein. By contrast, the difference in the reactivity between the two toxins (MAbs 5B4F11, 5C11E6, 4E10F8, 4E10E10, 14G2D7, 14G2G9, 11G2G9B10, 14G2B11F8, 14G2E11C7, 14G2E11B8, 14G2E11G8 and 23E6C8) might be related to the epitope/s lying in a variable region and/or with the high avidity of the MAbs versus recombinant CPB2 with respect to the wild-type form. Indeed, the MW of CPB2 expressed in insect cells is slightly higher than that of CPB2 protein expressed in bacteria (Fig. 4); therefore, the antigenic characteristics between the two toxins might also be slightly different with greater MAb avidity against recombinant CPB2. Finally, in the ELISA, the reactivity of the MAbs 5C11E6, 5B4F11, 4E10F8, 4E10E10 and 23E6C8 presented a high OD_450_ value for both recombinant and wild-type CPB2, whereas their reactivity in Western blotting was quite limited, indicating the role of structural conformation in stabilizing the interaction between the antigens and antibodies.

## Conclusions

In this study, we report the expression of deleted atypical CPB2 recombinant protein in a baculovirus system. High amounts of purified product were obtained applying a fast and simple method such as Ni^2+^ affinity chromatography. Interestingly, the demonstration of the non-toxicity of the deleted purified recombinant CPB2, allowed its utilization for the production of polyclonal antibodies and a battery of MAbs with different reactivities against recombinant and wild-type CPB2. The high degree of sensibility and specificity showed by these molecules in our assays, lays the groundwork for the standardization of an enzyme immunoassay. Moreover, these reagents could be used in further studies to understand the biological activity of atypical CPB2 toxin.

Finally, the high immunogenic power of this protein, associated to non-toxicity, might pave the way to its use for the development of a vaccine.

## Methods

### Cell culture, growth conditions and DNA extraction

*Clostridium perfringens* strains were isolated from cattle with enteritis and were genotyped by PCR. Two strains (6069 and 6157) belonging to the type A atypical *cpb*2+ genotype were chosen and were used to amplify the gene encoding CPB2 toxin. The strains were grown in agar plates (containing 5% of mutton blood), and subsequently, a single colony was transferred to cysteine broth (0.1% yeast extract, 27 mM glucose, 16 mM cysteine, 17 mM Na_2_HPO_4_·2H_2_O, 80 mM NaCl, 2% vegetable peptone). The bacteria were incubated anaerobically at 37 °C for 24 h and were subjected to DNA extraction after centrifugation at 16,000×*g* for 10 min using the QIAamp^®^DNA Mini Kit (QIAGEN). The extracted DNA was stored at −20 °C until use.

For wild-type CPB2 toxin production, the *cpb*2+ genotype *C. perfringens* strains were grown in TPGY medium (2% yeast extract, 22 mM glucose, 5% tryptone; 0.5% meat peptone). After 48 h, the cultures were centrifuged at 16,000×*g* for 10 min, and the supernatant was stored at −20 °C until use in the immunoenzymatic test.

### PCR amplification and molecular cloning

The genes encoding full-length and deleted atypical CPB2 proteins were generated using the oligonucleotides reported in Table 1. To obtain the deleted forms, amino acids from 1 to 25 of the putative signal sequence [6, 7, 23] were removed using PCR. The reactions were performed using an initial denaturing step at 95 °C for 2 min, followed by 35 amplification cycles of 95 °C for 15 s, 55 °C for 30 s and 68 °C for 1 min, and a final step at 68 °C for 5 min. The amplified products were analysed by 1% agarose gel electrophoresis and were purified using the QIAquick PCR purification Kit (QIAGEN). The purified amplicons were subjected to restriction digestion with *Eco*RI and *Age*I (New England Biolabs) followed by ligase reaction into the pOET2_C-6xHis baculovirus transfer vector (OXFORD Expression Technologies) in frame with the 6xHis-tag. The resultant constructs were verified by restriction analysis and sequencing using the BigDye Terminator v3.1 Cycle Sequencing kit (Applied Biosystems).

**Table 1:**
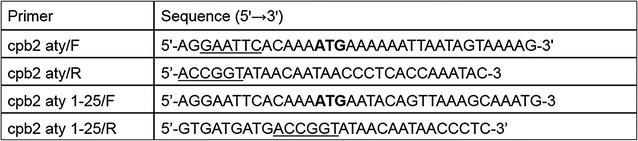
Nucleotide sequences of the primers used for the amplification of the full-length and deleted forms of CPB2 toxin

The restriction sites are underlined with a solid line, and ATG start codons are in bold

### Generation of recombinant baculovirus and expression of recombinant CPB2 proteins

The recombinant baculoviruses were obtained by co-transfection of Sf21 insect cells with the constructs containing the full-length and deleted atypical *cpb*2-amplified genes and linear *flash* **BAC** DNA as reported in the manufacturer’s instructions of the FlashBac system (Oxford Technologies Expression). After 7 days, the cellular supernatants, containing the recombinant baculoviruses, were harvested and used to prepare a high-titre stock. Recombinant protein production was performed by infection of Sf21 cells, grown in roller bottles in HYQ SFX serum-free medium (HyClone) with a 0.1- to 1-unit-forming plaque of recombinant baculovirus per cell. Three days post-infection, the infected SF21 cells and culture medium fractions were harvested and stored at −20 °C until use.

### Western blotting

Infected Sf21 cells were treated with lysis buffer (0.1 M NaH_2_PO_4_/Na_2_HPO_4_ at pH 8, 8 M urea, 5 mM DTT and protease inhibitor) and were sonicated for 3 cycles of 15 s with 2 min of rest. The samples were centrifuged at 16,000×*g* for 10 min, and the cellular lysates were recovered. The cellular lysates and supernatant containing CPB2 recombinant proteins were mixed with 4xNuPAGE sample buffer containing 10 mM DTT and were denatured at 95 °C for 5 min. The proteins were resolved by SDS-PAGE in 12% acrylamide pre-cast NuPAGE gels and then were transferred onto a PVDF membrane. The membranes were incubated with TBST blocking solution (20 mM Tris–HCl, 150 mM NaCl, 0.05% Tween 20 (v/v) at pH 7.4) containing 5% dry milk for 2 h at room temperature (RT), followed by overnight (ON) incubation with anti-6xHis-HRP MAb (Invitrogen). The immune reactions were visualized by chemiluminescence using the Super Signal West Pico Substrate kit (Thermo Fischer Scientific).

The reactivity of polyclonal serum and MAbs was verified by immunoblotting assay using recombinant and wild-type CPB2 toxins as antigens. Briefly, samples containing wild-type and recombinant atypical CPB2 (0.2 μg) toxins were resolved by SDS-PAGE as reported above. The proteins were transferred onto a nitrocellulose membrane using the iBlot^®^ Dry Blotting System (Invitrogen). The membrane was blocked with PBST containing 5% dry milk for 2 h at RT and, after three washes with PBST, was incubated ON at 4 °C with the MAbs supernatant. After three further washes, the membrane was incubated for 1 h at RT with an anti-mouse IgG-HRP-conjugate MAb (GE Healthcare). The immunoreactions were visualized using the ECL Select™ Western Blotting Detection Reagent (GE Healthcare), and image acquisition was carried out with Chemidoc MP (BIO-RAD). The same procedure was used to characterize polyclonal serum except for the secondary antibody, where the antigen–antibody reaction was carried out using an anti-rabbit HRP-conjugate MAb (GE Healthcare).

### Purification of recombinant deleted atypical CPB2 proteins

The purification of recombinant deleted atypical CPB2 toxin was performed using Ni^2+^ affinity chromatography (SIGMA-ALDRICH). Infected insect cells (1.5 × 10^9^) were treated with lysis buffer as previously reported; the cellular extract, after the addition of 20 mM imidazole, was subjected under denaturing conditions to Ni^2+^ affinity chromatography. Purified recombinant proteins were eluted using the elution buffer at pH 4.5 in accordance with the manufacturer and were dialysed against PBS buffer ON at 4 °C using the slyde-A-lyzer G2 dialysis cassettes with a size of 3.5 K MCWO (Thermo Fischer Scientific). The proteins were quantified by the Bradford method (BIO-RAD), and the purity was determined by staining the SDS-PAGE gel with the GelCode Blue Staining Reagent (Thermo Fisher Scientific).

### In vitro and in vivo toxicity tests

The in vitro toxic activity of full-length and deleted CPB2 recombinant proteins was verified using the CaCo2 cell line [Caco2] (ATCC^®^ HTB-37™). Briefly, both recombinant proteins were diluted in Eagle’s Minimum Essential Medium supplemented with 10% foetal bovine serum (FBS) at two different concentrations (10 µg/ml and 50 µg/ml) and were added to confluent CaCo2 cells in 48-well plates (0.5 ml per well for both dilutions). The cells were incubated at 37 °C and were inspected microscopically after 5, 12 and 24 h. As a negative control, CaCo2 cells were cultured with only medium.

The in vivo toxicity test was performed by inoculating 100 μg of purified recombinant deleted atypical CPB2 toxin into laboratory animals in accordance with the Italian Official Pharmacopoeia (XII Ed.) [27].

### Polyclonal and monoclonal antibody production

The purified recombinant CPB2 protein was used to immunize white rabbits and Balb/c mice to obtain polyclonal antiserum and monoclonal antibodies, respectively. The animals were injected intraperitoneally; the first two inocula were carried out with the additions of Freund’s complete and incomplete adjuvant (SIGMA-ALDRICH), whereas the subsequent injections were performed with sterile PBS. Three days after the last injection, immune sera were collected and tested by undirected ELISA to verify the seroconversion. Rabbit immune sera containing polyclonal antibodies were collected and stored at −20 °C until use. Immunized Balb/C mice were used to generate monoclonal antibody-producing hybridoma cell lines as reported previously [30]. Briefly, splenocytes were collected and fused with the murine Sp2/O-Ag-14 myeloma cell line (ATCC CRL-1581™). The hybridomas obtained were cultured in Dulbecco’s Modified Eagle’s Medium containing 20% foetal bovine serum (SIGMA-ALDRICH) and HAT Media supplement 50× (HybriMax^®^, SIGMA-ALDRICH) and were screened by indirect ELISA (iELISA). Hybridomas with values of optical density at 450 nm (OD_450 nm_) ≥0.300 were considered positive and were cloned according to the method of limiting dilutions [28–30].

### Indirect ELISA (iELISA)

Ninety-six-well microplates (PolySorp, Nunc International, Denmark) were coated with deleted recombinant atypical CPB2 toxin, diluted in 50 mM carbonate–bicarbonate buffer pH 9.6, to a final concentration of 10 μg/ml per well and were incubated ON at 4 °C. After blocking with 1% yeast extract in PBS containing 0.05% Tween 20 (PBST), hybridoma supernatants were added into the wells, and the plates were incubated for 1 h at 37 °C. After washing with PBST, HRP-conjugated goat anti-mouse IgG (GE Healthcare) was added, and the plates were incubated for 30 min at RT. After further washings, 100 μl/well of the chromogen substrate 3,3′, 5,5′-tetramethylbenzidine (TMB) (SIGMA-ALDRICH) was added to each well for 20 min. The reaction was stopped with H_2_SO_4_ 0.5 M, and the OD_450_ was measured.

The MAb isotype was determined using the Mouse-Typer^®^ Isotyping Panel kit (BIO-RAD) according to the manufacturer’s instructions.

### ELISA sandwich

The reactivity of MAbs as a revelatory system was tested by ELISA using polyclonal antibodies as the catcher. Briefly, ninety-six-well MaxiSorp microplates (MaxiSorp Nunc International, Denmark) were coated with polyclonal antibody diluted from 1:10,000 to 1:100,000 in carbonate–bicarbonate buffer, pH 9.6, at +4 °C ON. After three washes with PBST, *C. perfringens* culture medium containing wild-type and recombinant atypical CPB2 toxins (diluted 1:4 in PBST) was added and incubated for 1 h at 37 °C. The plates were further washed three times, and subsequently, the specific MAb growing medium (serial dilutions from 1:5 to 1:640) was added. After an additional incubation for 1 h at 37 °C and washes, the plates were incubated with anti-mouse HPR-conjugated MAb (GE Healthcare). Next, the TMB chromogenic substrate was added to the plates and was incubated for 15 min at RT. The reaction was stopped with 0.5 M H_2_SO_4_, and the OD_450_ nm was measured. In addition, the same ELISA test, except for the use of an anti-rabbit as a revelatory system, was employed to evaluate the reactivity of MAbs as capturing molecules.

Finally, to establish the specificity of the MAbs, C*. perfringens* culture medium containing wild-type consensus CPB2 (diluted at 1:4 in PBST) was used as antigen in the same ELISA with the exception of the polyclonal antibody concentration, which was coated at dilution 1:10,000.

## Abbreviations

CPB2: *C. perfringens* beta-2 toxin
MAb: monoclonal antibody
rCPB2_Δ1–25_-His_6_: deleted recombinant C-terminal 6xHis-tagged atypical CPB2 toxin
aa: amino acids
ELISA: Enzyme-linked Immunosorbent Assay
PCR: polymerase chain reaction
DTT: dithiothreitol
RT: room temperature
ON: overnight
SDS-PAGE: sodium dodecyl sulphate–polyacrylamide gel electhrophoresis
FBS: foetal bovine serum
iELISA: Indirect Enzyme-linked Immunosorbent Assay
TMB: 3,3′, 5,5′-tetramethylbenzidine
MW: molecular weight
CPE: *C. perfringens* enterotoxin
